# Early detection of cognitive impairment in end-stage renal disease patients undergoing hemodialysis: insights from Resting-State functional connectivity analysis

**DOI:** 10.1186/s12882-025-04109-z

**Published:** 2025-04-14

**Authors:** Mengchen Liu, Rujin Li, Man Liang, Jiejing Li, Shandong Meng, Weizhao Lin, Zhihua Zhou, Kanghui Yu, Yanying Chen, Yi Yin, Shoujun Xu, Wenqing Xiao, Zichao Chen, Guihua Jiang, Yunfan Wu

**Affiliations:** 1 Department of Medical Imaging, The Affiliated Guangdong Second Provincial General Hospital of Jinan University, Guangzhou, PR China; 2 The Second School of Clinical Medicine, Guangdong Second Provincial General Hospital, Southern Medical University, Guangzhou, PR China; 3 The Department of Nuclear Medicine Department, The Affiliated Guangdong Second Provincial General Hospital of Jinan University, Guangzhou, PR China; 4 The Department of Renal Transplantation, The Affiliated Guangdong Second Provincial General Hospital of Jinan University, Guangzhou, PR China; 5 Department of Radiology, Jieyang People’s Hospital, Jieyang, PR China; 6 Department of Neurology, The First Affiliated Hospital/School of Clinical Medicine of Guangdong Pharmaceutical University, Guangzhou, PR China; 7 Department of Medical Imaging, Guangdong Second Provincial General Hospital, School of Medicine, Jinan University, Guangzhou, PR China; 8 Department of Radiology, Shenzhen Children’s Hospital, Shenzhen, PR China

**Keywords:** End stage renal disease, Hemodialysis, Cognitive impairment, Identify, Independent component analysis, Functional connectivity

## Abstract

**Background:**

This study aims to investigate the characteristics of functional connectivity (FC) in neurologically asymptomatic patients with end-stage renal disease (ESRD) undergoing hemodialysis (HD) and experiencing cognitive impairment (CI).

**Methods:**

36 early-stage ESRD patients undergoing HD (ESHD) and 31 healthy control subjects underwent MRI scans. Abnormal FCs and networks were identified between the two groups, and correlation analysis and Area Under the Curve (AUC) analysis were conducted between abnormal FC regions and clinical variables.

**Results:**

The ESHD group exhibited abnormal FCs in the posterior default mode network (DMN), attention network, and external visual network (VN). Significant correlations were observed between FC values of multiple brain regions and neurocognitive scores in the ESHD group. Additionally, the FC value of the right median cingulate gyrus negatively correlated with serum calcium levels. AUC analysis demonstrated that altered FC values in the left angular gyrus and the right supramarginal gyrus effectively distinguished patients with or without CI.

**Conclusions:**

In conclusion, our study reveals multiple abnormal FC regions in asymptomatic ESHD patients, affecting visual-spatial processing, short-term memory, language, attention, and executive function. Altered FCs and their negative correlation with serum calcium levels highlight a potential link between metabolic disturbances and cognitive decline, suggesting new opportunities for targeted interventions in this vulnerable population.

## Introduction

Chronic kidney disease (CKD) represents a significant global public health concern. The advanced stage of CKD, known as end-stage renal disease (ESRD), stands as a primary contributor to morbidity and mortality worldwide [1]. Projections indicate that the population requiring hemodialysis (HD) as replacement therapy will reach 14.5 million by 2030 [1]. Notably, compared to patients with chronic kidney disease (CKD) or those undergoing peritoneal dialysis (PD), a prospective study suggested that 79.4% of individuals undergoing hemodialysis (HD) exhibit heightened susceptibility to cognitive impairment [2], encompassing conditions such as dementia and Alzheimer’s disease (AD) [3]. ESRD patients undergoing HD who experience cognitive impairment face elevated risks of disability and mortality [4]. Particularly, individuals with ESRD undergoing HD face a significant risk of developing dementia and AD, with these diagnoses being associated with a twofold increase in mortality rates [3]. In summary, the co-occurrence of cognitive impairment in ESRD patients entails increased financial burdens on governments, communities, and families, as well as reduced productivity [4]. Furthermore, HD-related cognitive impairment may result from acute and chronic brain injuries induced by exacerbated cerebral edema, fluctuations in cerebral blood flow, and osmotic shifts [5]. Nevertheless, the neurobiological mechanisms underpinning HD-related cognitive impairment remain unclear [6]. Therefore, the early detection of brain abnormalities in neurologically asymptomatic patients undergoing maintenance HD is imperative for prompt diagnosis, intervention, and ultimately, the mitigation of mortality rates. Additionally, there is currently no effective screening method for identifying asymptomatic ESRD patients undergoing HD with cognitive impairment during the early stages of hemodialysis in clinical practice.

Resting-state functional connectivity (FC) analysis serves as a noninvasive approach for estimating alterations in neural interactions among brain regions. Independent component analysis (ICA) represents a blind source separation technique employed to discern FC changes within brain-wide resting-state networks (RSNs) associated with cognitive tasks, without the need for a priori selection of seed regions. ICA has been extensively utilized in investigating various neurological and psychiatric disorders, including epilepsy [7], schizotypal personality disorder [8], and autism spectrum disorders [9]. In a study by Chen et al. [10], ICA was employed to compare RSN changes between 22 healthy control (HC) subjects and 37 patients with end-stage renal disease (ESRD) before and after kidney transplantation. The findings revealed enhanced FC within the default mode network (DMN) and sensorimotor network (SMN) in patients with ESRD six months post-transplantation. This research underscores the potential of ICA in identifying abnormal brain function within RSNs in ESRD patients, offering objective imaging evidence to elucidate the underlying clinical symptoms associated with FC alterations in the hemodialysis (HD) group. However, despite the increasing recognition of cognitive impairment in the HD population, it remains unclear which specific brain regions exhibit abnormal FC within RSNs during the initial year of dialysis in patients with end-stage renal disease undergoing hemodialysis (ESHD). Furthermore, to the best of our knowledge, no studies have yet demonstrated the utility of FC analysis using ICA as a screening tool for identifying asymptomatic ESHD patients with cognitive impairment during the early stages of hemodialysis.

It has been reported that the reorganization of brain structure is intricately associated with the pathophysiology of ESRD [11]. Consequently, we hypothesize that individuals with end-stage renal disease (ESRD) undergoing hemodialysis (ESHD) may experience alterations in functional connectivities (FCs) as detected by the ICA technique. We posit that this method holds significant promise for assessing early-stage changes in asymptomatic ESHD patients with cognitive impairment. By elucidating these FC characteristics, our goal is to gain insights into the underlying mechanisms of cognitive impairment in ESHD. Furthermore, we propose that these FC alterations may serve as a potential screening method for identifying early asymptomatic ESHD patients at risk of cognitive impairment.

## Materials and methods

### Subjects

Approval for this study was obtained from The Affiliated Guangdong Second Provincial General Hospital of Jinan University Human Research Ethics Committee, and informed consent was obtained from each participant. Between 2016 and 2021, a total of 36 newly initiated end-stage renal disease (ESRD) patients undergoing hemodialysis (ESHD) were recruited from the renal transplantation department of the Affiliated Guangdong Second Provincial General Hospital of Jinan University. These patients had been undergoing dialysis for durations ranging from 18 days to 12 months, with sessions conducted 3–4 times per week. Additionally, 31 healthy control (HC) subjects matched for age, sex, and education level were recruited from the community. Inclusion criteria stipulated that participants be between 20 and 65 years of age and right-handed.

All patients diagnosed with end-stage renal disease (ESRD) underwent evaluation by two physicians with at least 15 years of experience. ESRD, classified as stage 5 chronic kidney disease (CKD), was determined according to the K/DOQI classification, with a glomerular filtration rate (GFR) of less than 15 ml/min/1.73 m^2. The primary cause of ESRD in all patients was chronic glomerulonephritis. Patients with ESRD received conventional hemodialysis (HD) treatment. Furthermore, all patients were asymptomatic and had no history of neurologic or psychiatric diseases. The etiology and duration of HD were obtained from the patients’ electronic medical records. Exclusion criteria included a history of drug or alcohol abuse, known severe neurological disorders (such as stroke and epilepsy), psychiatric disorders, traumatic brain injury, obvious brain lesions, or intolerance to magnetic resonance imaging (MRI).

### Neurocognitive testing

Before acquiring MRI data, all participants underwent a battery of neuropsychological tests, including the following: (1) Digital Connection Test Types A and B (NCT-A/B): Assessing visual perception and motor speed abilities (NCT-A) and visual perception, working memory, attention, and executive control (NCT-B). (2) Line-Tracing Test (LTT): Evaluating visual discrimination and attention. (3) Serial Dot Test (SDT): Measuring spatial ability, reaction speed, and fine motor skills. (4) Digit Symbol Test (DST): Testing short-term memory, reaction speed, and attention accuracy. (5) Mini-Mental State Examination (Folstein version): A widely used screening tool for cognitive impairment. (6) Montreal Cognitive Assessment (MoCA): Another screening tool assessing various cognitive domains. NCT-A/B, DST, SDT, and LTT tests primarily assess attention, psychomotor speed, visual memory, and other cognitive functions [12]. Cognitive impairment in ESHD cases was diagnosed by two neurologists with 15 years of experience.

### Laboratory tests

All patients with end-stage renal disease undergoing hemodialysis (ESHD) underwent blood biochemical examinations, including tests for hemoglobin, serum calcium, serum kalium, serum urea nitrogen, and serum creatinine, within 24 h of MR examination. Notably, blood laboratory tests were not performed on the healthy control (HC) group. All blood samples were obtained, and MR data were acquired prior to dialysis.

### MR data acquisition

MRI scans were conducted for all patients with end-stage renal disease (ESRD) and HC subjects using a Philips Ingenia 3.0T MR scanner system equipped with a 32-channel phased-array head coil at the Department of Medical Imaging, Guangdong Second Provincial General Hospital. Participants were positioned supine and securely immobilized during scanning, as previously described [13].

For functional MRI (fMRI), echo-planar imaging (EPI) was employed with the following parameters: axial slices, 33; repetition time (TR), 2000 ms; echo time (TE), 30 ms; flip angle (FA), 90°; slice thickness, 3.5 mm without gaps; matrix size, 64 × 64; and field of view (FOV), 230 mm × 230 mm^2^; voxel size, 3 × 3 × 3 mm^3^. Each participant underwent acquisition of 240 volumes during the resting-state fMRI scan. Further, individual three-dimensional T1-weighted images (T1WI) were acquired by a Fast-field echo (FFE) pulse sequence via the following parameters: 160 sagittal slices; TR, 25 ms; TE, 4.1 ms; FA, 30º; slice thickness, 1.0 mm with no gap; field-of-view (FOV), 230 × 230mm^2^; voxel size, 1 × 1 × 1 mm^3^, and matrix, 230 mm × 230 mm, total volume of 240. Each resting-state fMRI scan lasted 8 min.

Following the completion of the scan, all subjects underwent an interview to assess their level of cooperation. Additionally, each subject underwent a routine MRI sequence aimed at detecting any underlying diseases, which included acquiring T1-weighted images (T1WI) and T2 Fluid Attenuated Inversion Recovery (FLAIR) sequences. The acquired conventional MRI images were independently reviewed by two physicians, each possessing at least 15 years of experience. Importantly, the reviewing physicians were blinded to whether the images belonged to a patient or a healthy control subject.

### Imaging analysis

All resting-state fMRI datasets were preprocessed using the DPARSF 5.3 Advanced Edition plugin software within DPABI 6.2_220915 (http://rfmri.org/dpabi) [14]. The initial 10 images were discarded to eliminate potential interference and ensure steady-state longitudinal magnetization. Subsequently, the remaining 230 images underwent correction for temporal differences and head motion. A movement threshold of head motion > 1.0 mm or 1.0° was applied; however, no subject was excluded as head movements did not exceed the threshold. For spatial normalization, the standard Montreal Neurological Institute (MNI) template was utilized with the DARTEL method at a resolution of 3 × 3 × 3 mm^3^. Initially, each subject’s T1 image was co-registered with their respective functional images. Next, the DARTEL method segmented the T1 image into gray and white matter, along with cerebrospinal fluid. Subsequently, the segmented gray matter probability map underwent spatial transformation. The resulting image transformation parameters were then applied to the corresponding fMRI functional images for spatial standardization. Following spatial normalization, linear trends were filtered using a typical temporal bandpass (0.01–0.08 Hz). Subsequently, a Gaussian kernel with a full width at half maximum of 8 mm was applied to spatially smooth the resulting time series. Two radiologists with ≥ 15 years of experience reviewed the images to ensure segmentation quality and registration.

### Group ICA analysis

**Fig. 1:**
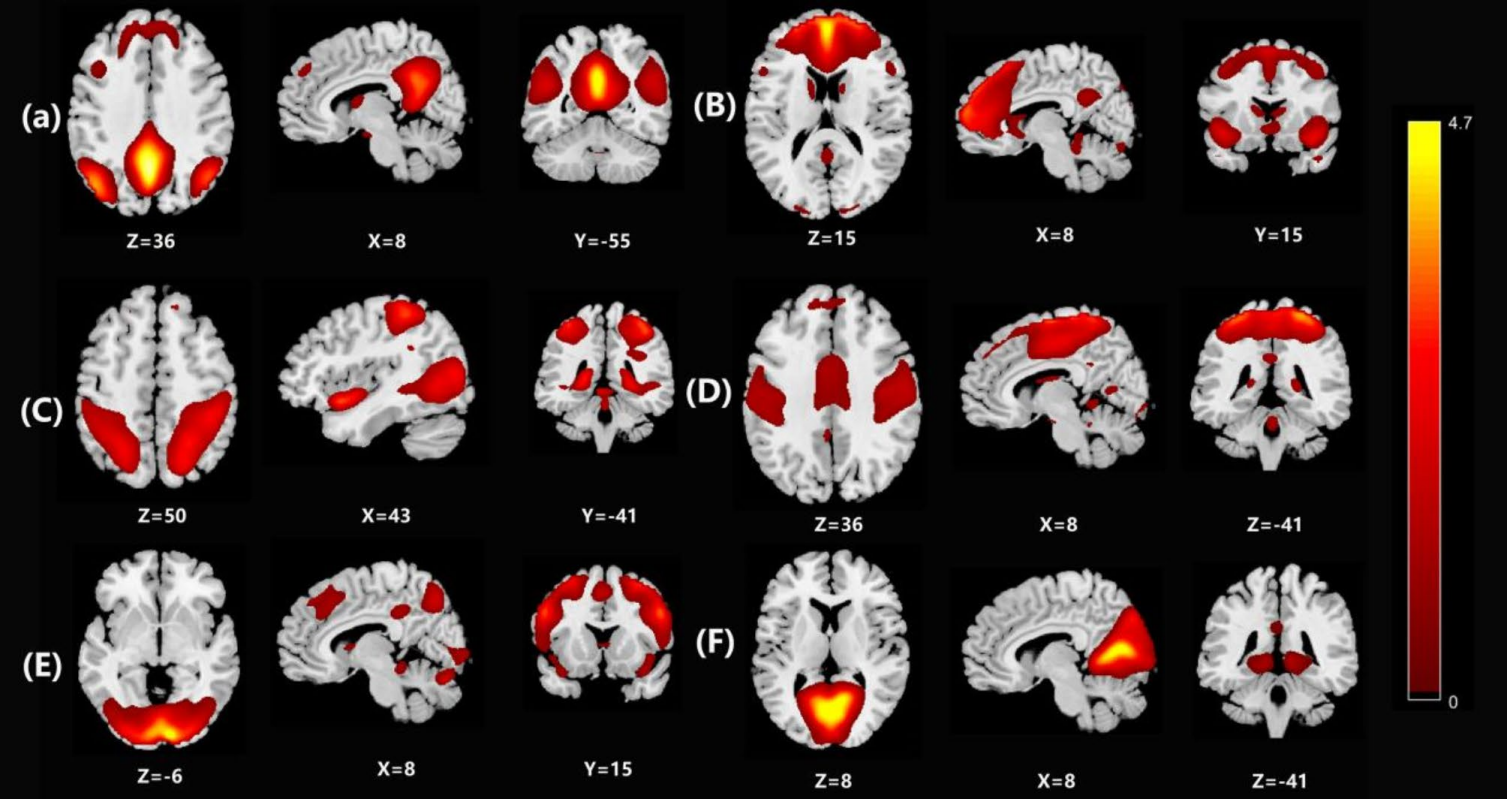
The six resting-state networks which cross-correlated were identified for all subjects: (**A**) the posterior Default Mode Network (pDMN), (**B**) the anterior Default Mode Network (aDMN), (**C**) the attention network (AN), (**D**) the sensorimotor network (SMN), (**E**) the external visual network (EVN), (**F**) the lateral visual network (LVN). Coordinates (x y z) refer to MNI space

All pre-processed images underwent analysis using Group ICA (GIFT) from the fMRI Toolbox to delineate large-scale patterns of functional connectivity (FC). The ICA analysis comprised three stages for each subject: (a) data dimensionality reduction, (b) application of the ICA algorithm, and (c) reverse reconstruction. Utilizing the automatic score estimation feature of the GIFT software, we employed ICA to reduce the dimensions of all subjects’ fMRI images to 24 spatial components. Subsequently, we computed the cross-correlations of these 24 networks with a well-defined template. Based on the brain network template established by Smith et al. [15], we identified six brain resting-state networks (RSNs) of interest for all subjects through correlation order and visual inspection: the anterior default mode network (aDMN), posterior default mode network (pDMN), sensorimotor network (SMN), attention network (AN), lateral visual network (LVN), and external visual network (EVN) (Fig. 1).

### Statistical analysis

Comparative analysis of demographic and clinical characteristics was performed using SPSS 20.0 software (SPSS Inc., Chicago, USA). Initially, the demographic and clinical characteristics of hemodialysis (HD) and healthy control (HC) subjects were compared using the Mann-Whitney U test. Data normality was verified using the Shapiro-Wilk test. Additionally, a chi-square test was employed to compare intergroup qualitative variables, such as gender. Intergroup differences in age, education, neuropsychological test scores, and clinical laboratory indicators were assessed using two-sample t-tests.

Consistent with a prior study [8], a one-sample t-test was employed to analyze the resting-state networks (RSNs) within each group and identify significant clusters, with statistical significance set at *P* < 0.05 after family-wise error (FWE) correction. Subsequently, a combination map of the hemodialysis (HD) and healthy control (HC) groups was utilized to mask the group analysis of each RSN component. This approach ensured that only alterations relevant to each RSN were analyzed. Two-sample t-tests were then conducted to explore functional connectivity (FC) based on each spatial component of the six RSNs identified through Group ICA. Clusters exceeding 20 voxels and exhibiting *P* < 0.05 with FWE correction were considered statistically significant. Following intergroup comparisons, partial correlation analysis was conducted within the HD group, rigorously controlling for age, sex, and years of education as covariates to account for their potential confounding effects.

Receiver operating characteristic (ROC) curves were generated to assess the discriminative ability of each functional connectivity (FC) in distinguishing patients with cognitive impairment from those without cognitive impairment during early hemodialysis. The area under the ROC curve (AUC) was calculated and reported as AUC ± standard deviation (SD). Cutoff values were selected based on the highest Youden index, calculated as [1 − (1 − sensitivity) × (1 − specificity)]. Statistical significance was defined as probability (P) values less than 0.05. Data were presented as mean ± SD if normally distributed or as median (interquartile range) if not normally distributed.

## Results

### Demographic and clinical features

Thirty-six neurologically asymptomatic ESRD patients initiating HD treatment, with dialysis durations ranging from 18 days to 12 months and sessions conducted 3–4 times a week, were enrolled. Among them, 19 patients exhibited minimal to mild cognitive impairment, while 17 patients showed no cognitive impairment based on a battery of neuropsychological tests. Eighteen patients had diabetes, and thirty-four patients had hypertension. There were no significant differences observed in age, sex, education, or scores on the SDT, DST, LTT, and MMSE between the groups (all *P* > 0.05). However, the scores on the NCT-A/B were significantly higher in the ESHD group compared to the HC group (*P* < 0.05), and the MoCA score was significantly lower in the ESHD group compared to the HC group (*P* < 0.05) (Table 1).

**Table 1:** Demographics and clinical characteristics of all participants

Characteristic	Participants on during early hemodialysis (*n* = 36)	HC participants (*n* = 31)	*P* value	
Age (years)	44.33 ± 10.61	44.13 ± 10.63	0.685	
Sex (male/female)	25/11	18/13	0.444	
Duration of education (yeas)	10.4 ± 3.5	9.7 ± 4.1	0.417	
Hemodialysis duration (months)	13.4 ± 12.0	/	/	
MMSE (score)	28.17 ± 1.61	28.45 ± 1.75	0.106	
MoCA (score)	24.33 ± 3.94	27.45 ± 2.20	< 0.001	
NCT-A (s)	53.00 ± 27.81	45.90 ± 10.44	< 0.001	
NCT-B (s)	93.42 ± 54.67	77.16 ± 29.57	0.001	
DST (score)	45.36 ± 8.91	51.39 ± 5.94	0.331	
SDT (s)	60.81 ± 12.87	44.13 ± 10.73	0.186	
LTT (s)	50.72 ± 10.77	44.19 ± 8.78	0.110	
Hemoglobin (g/l)	94.00 ± 18.83	/	/	
Serum urea nitrogen (mmol/L)	25.24 ± 6.94	/	/	
Serum creatinine (µmol/L)	1012.23 ± 316.64	/	/	
Serum Kalium (mmol/l)	4.70 ± 0.68	/	/	
Serum Calcium (mmol/l)	2.19 ± 0.23	/	/	

Note. Unless otherwise noted, data are expressed as mean ± SD. MMSE = the Folstein version of the Mini-Mental State Examination; MoCA = the Montreal Cognitive Assessment; NCT-A/B = Digital connection test types A and B; DST = the digit symbol test; SDT = the Serial Dot Test; LTT = the Line Tracing Test; HC = healthy control

**Fig. 2:**
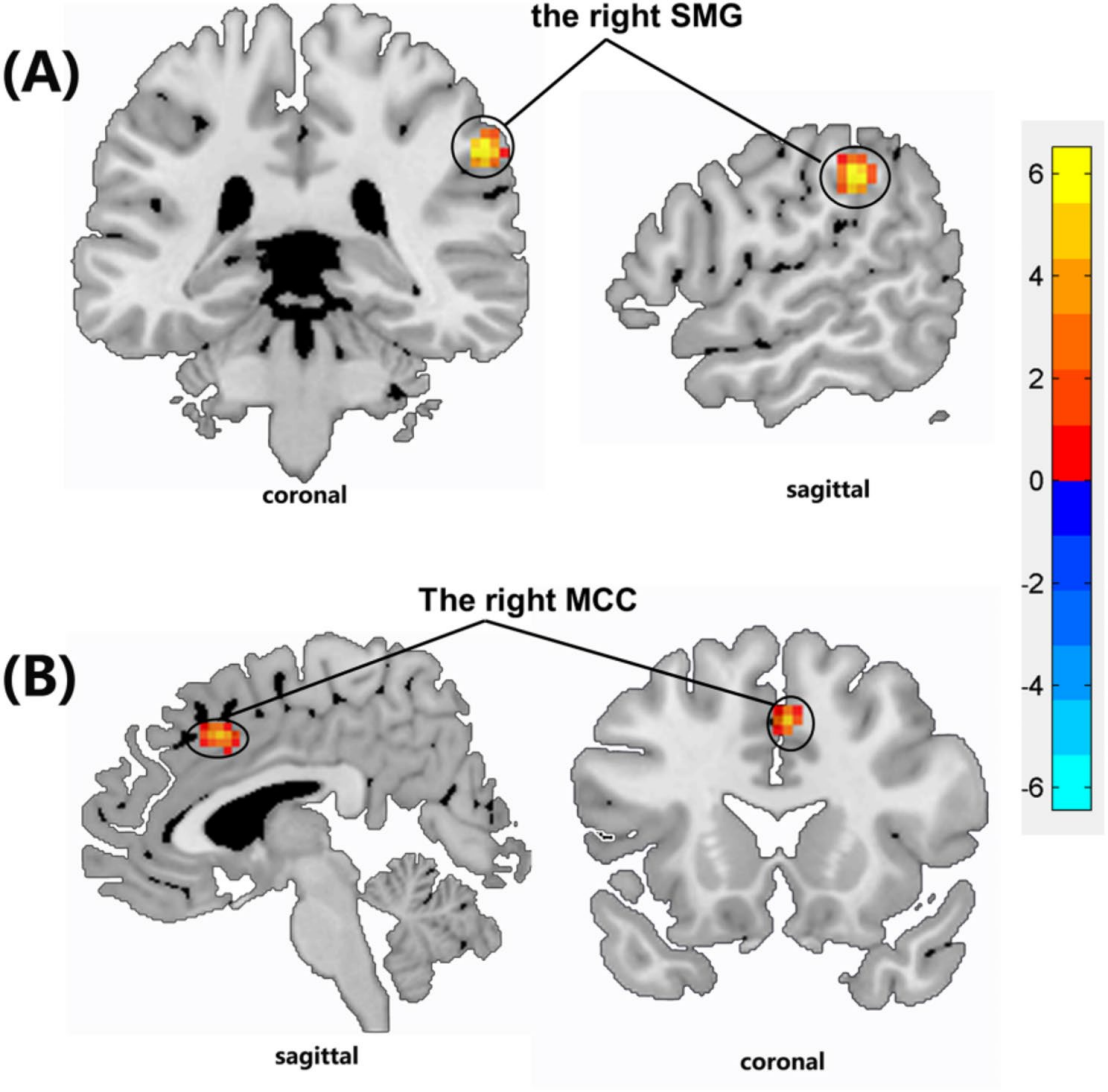
Brain regions showing increased functional connectivity (**A** and **B**) in End-stage renal disease patients on hemodialysis compared to those in healthy control subjects by two-samples t-test (*p* < 0. 05, family-wise error corrected). (**A**) SMG, supramarginal gyrus; (**B**) MCC, median cingulate gyrus

In comparison to the HC group, patients with ESHD exhibited aberrant FC in multiple brain regions within three RSNs: the pDMN, AN, and EVN. No significant differences were observed in the FC of the SMN between the ESHD and HC groups (*P* < 0.05, family-wise error corrected). Specifically, compared to the HC group, the FC of the right supramarginal gyrus (SMG) and right median cingulate gyrus (MCC) within the pDMN was increased in the ESHD group (*P* < 0.05, family-wise error corrected) (Fig. 2).

**Fig. 3:**
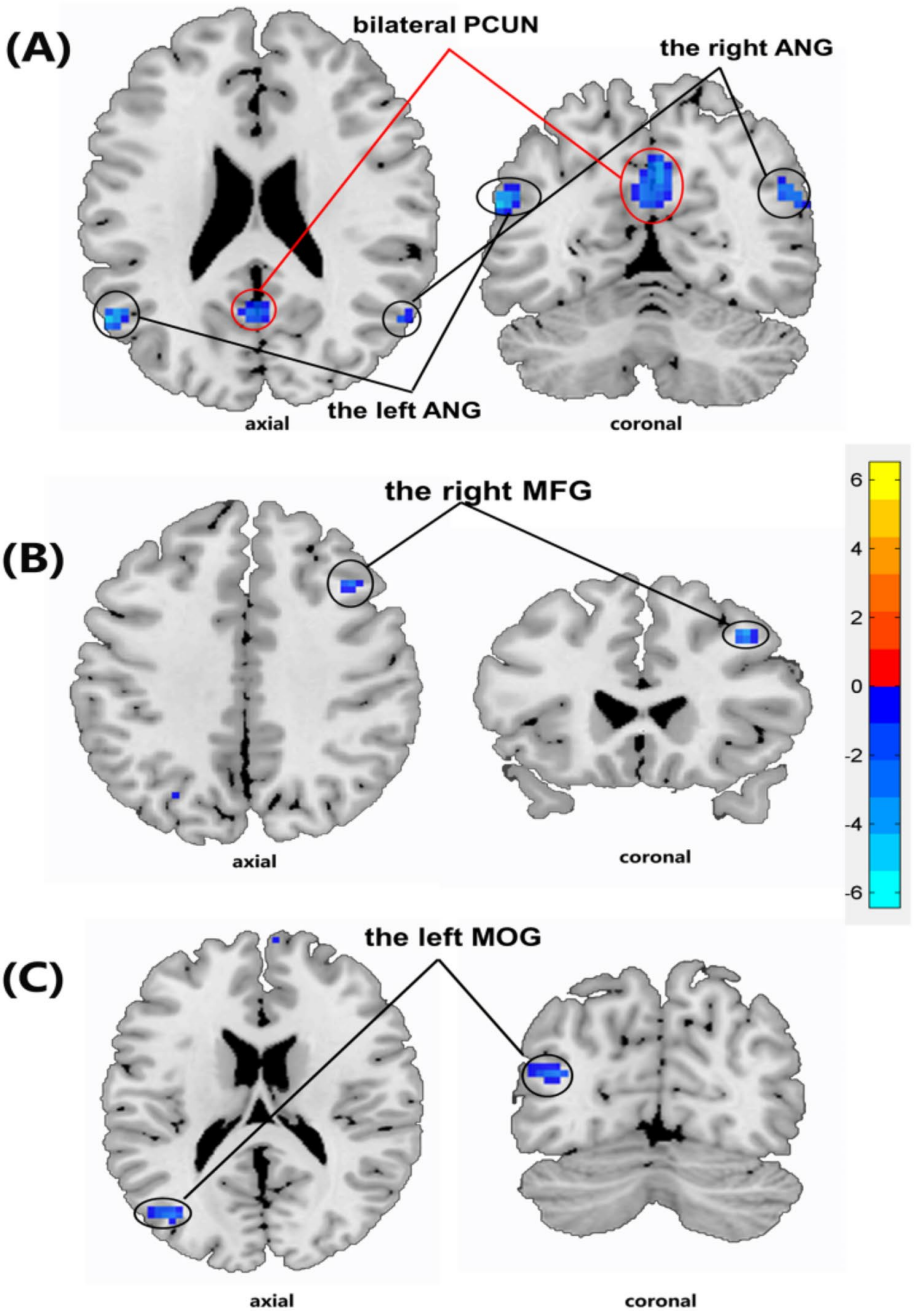
Brain regions showing decreased functional connectivity (**A, B** and **C**) in End-stage renal disease patients on hemodialysis compared to those in healthy control subjects by two-samples t-test (*p* < 0. 05, family-wise error corrected). (**A**) PCUN, precuneus gyrus; ANG, angular gyrus; (**B**) MFG, middle frontal gyrus; (**C**) MOG, middle occipital gyrus

Furthermore, decreased FC regions were identified within the pDMN, including the bilateral angular gyrus (ANG) and bilateral precuneus gyrus (PCUN) (*P* < 0.05, family-wise error corrected) (Fig. 3A). Additionally, reduced FC regions were observed in the right middle frontal gyrus (MFG) of the AN and the left middle occipital gyrus (MOG) of the EVN (*P* < 0.05, family-wise error corrected) (Fig. 3B and C).

**Fig. 4:**
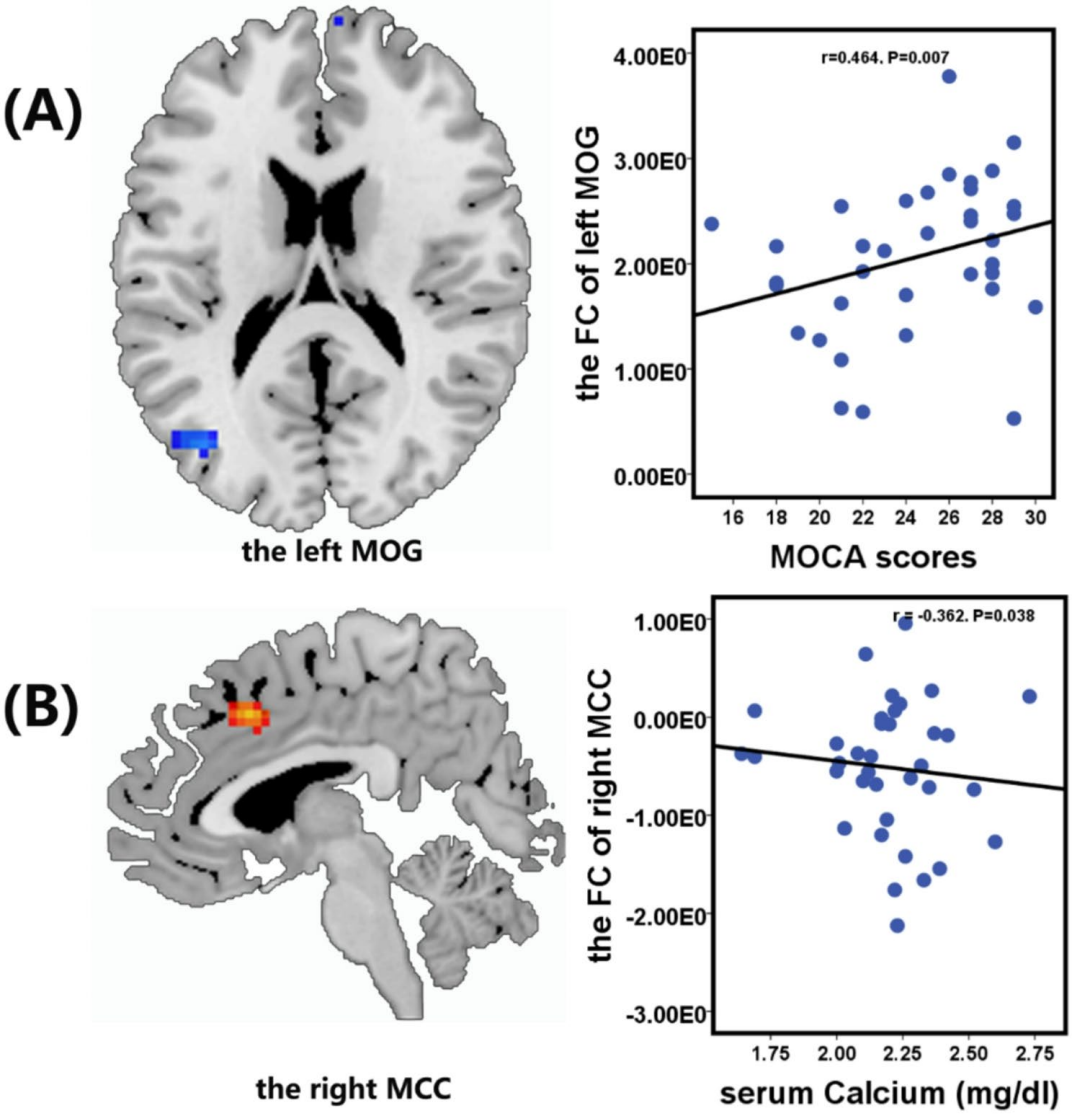
Significantly positive correlation between the functional connectivity value in the left middle occipital gyrus (MOG) and the Montreal Cognitive Assessment (MoCA) scores in End-stage renal disease patients on hemodialysis (**A**). Significantly negative correlation between the functional connectivity value in the right median cingulate gyrus (MCC) and serum calcium in End-stage renal disease patients on hemodialysis (**B**)

**Fig. 5:**
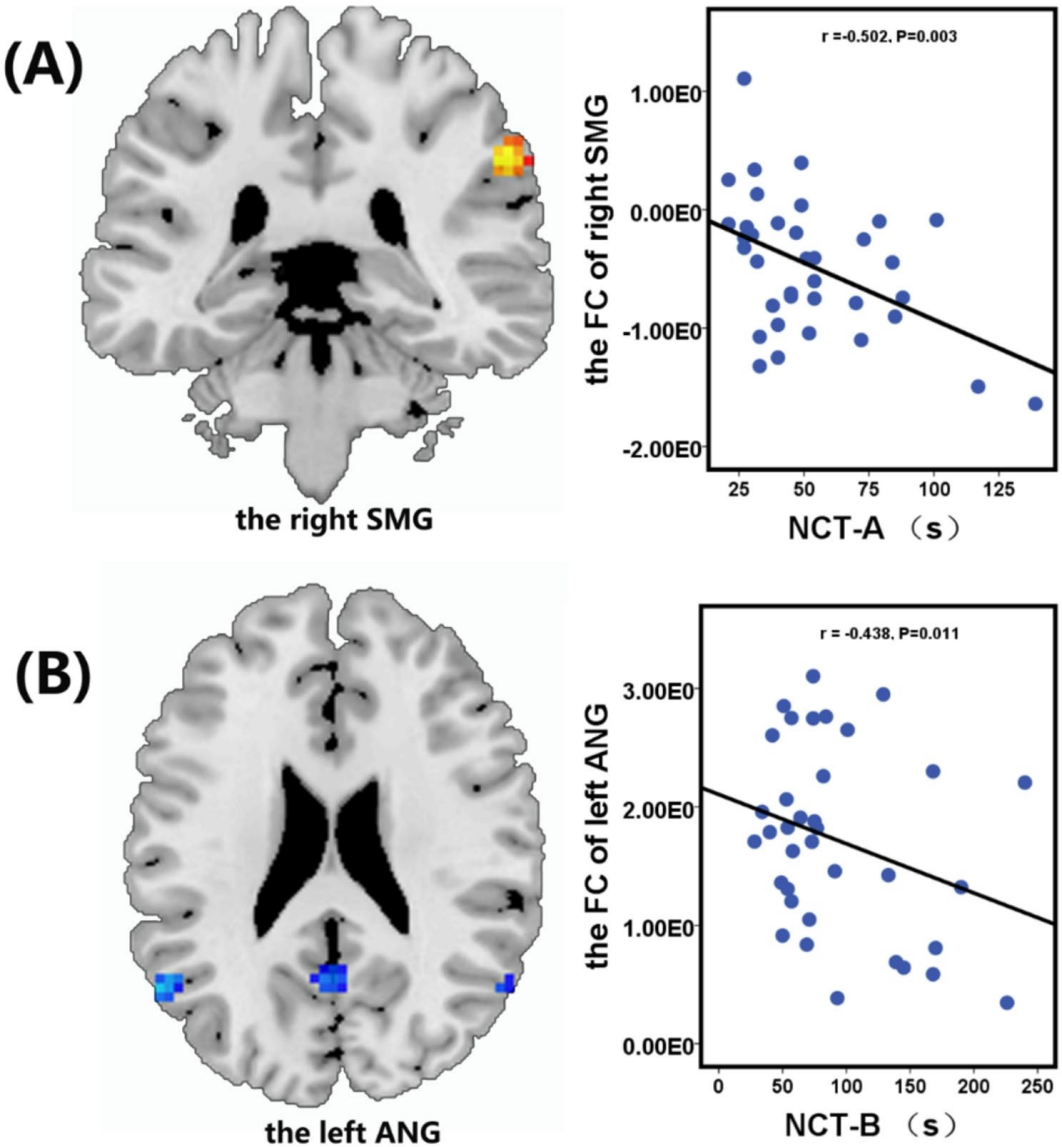
Significantly negative correlation between the functional connectivity value in the right supramarginal gyrus (SMG) and the Digital Connections Test Types A (NCT-A) scores in in End-stage renal disease patients on hemodialysis (**A**). Significantly negative correlation between the functional connectivity value in the left angular gyrus (ANG) and the Digital Connections Test Types B (NCT-B) scores in End-stage renal disease patients on hemodialysis (**B**)

Negative correlations were observed between the FC value of the right MCC and serum calcium level (*r* = -0.362, *P* = 0.038) (Fig. 4B), between the FC value of the right SMG and the NCT-A score (*r* = -0.502, *P* = 0.003) (Fig. 5A), and between the FC value of the left ANG and NCT-B score (*r* = -0.438, *P* = 0.011) (Fig. 5B). Additionally, a significant positive correlation was observed between the FC value of the left MOG and the MoCA score (*r* = 0.464, *P* = 0.007) (Fig. 4A). No significant correlations were found between FC values and scores on the MMSE, LTT, or DST.

**Fig. 6:**
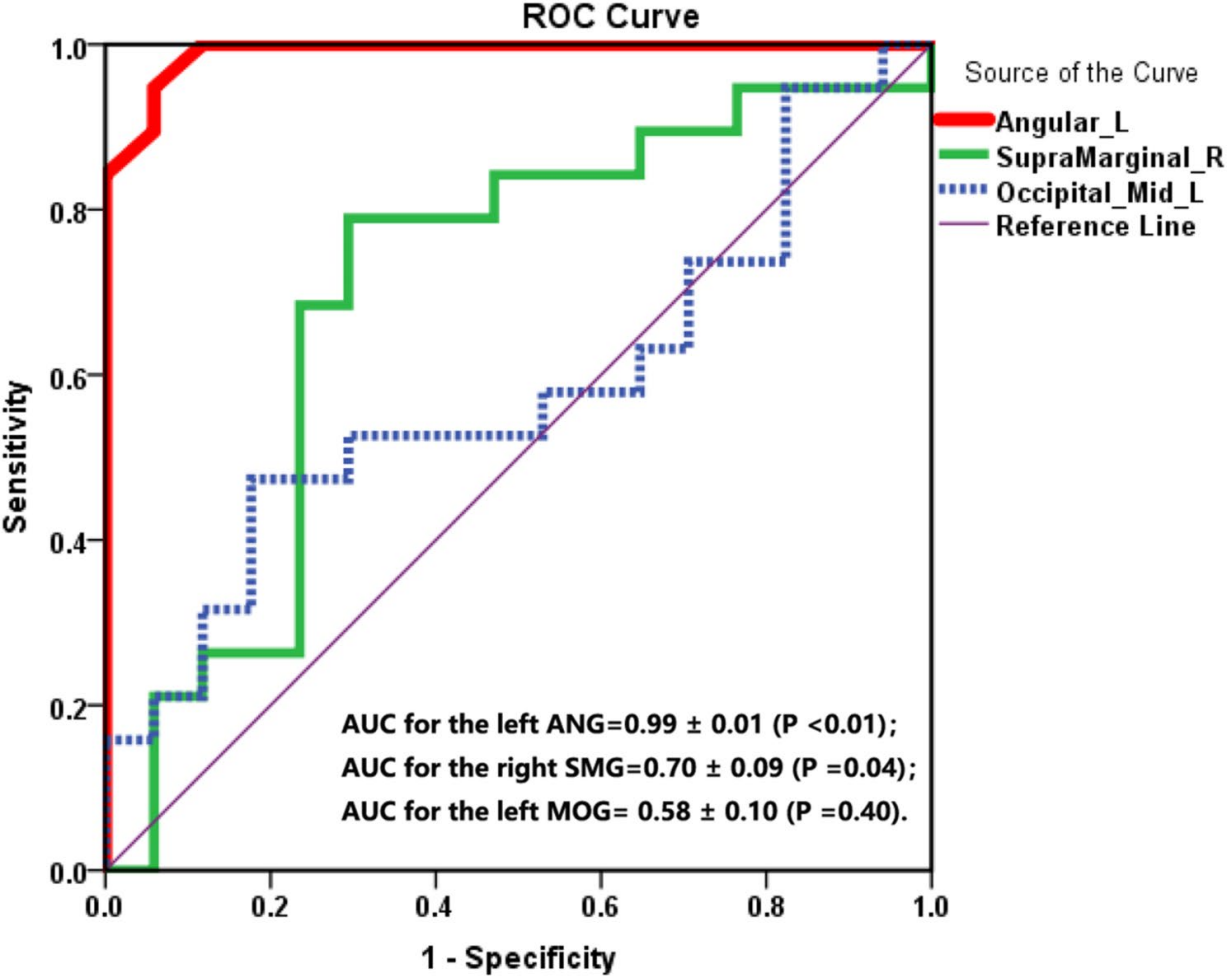
Receiver operator characteristic (ROC) curves for the functional connectivity value of the left angular gyrus (ANG), the right supramarginal gyrus (SMG) and the left middle occipital gyrus (MOG). The AUCs for the left ANG, the right SMG, and the left MOG were 0.99 ± 0.01 (*P* < 0.01), 0.70 ± 0.09 (*P* = 0.04), and 0.58 ± 0.10 (*P* = 0.40), respectively

As depicted in Fig. 6, the area under the curve (AUC) values for the FC value of the left ANG and the right SMG suggest that both can accurately distinguish patients with cognitive impairment from those without cognitive impairment during early hemodialysis. The AUC values for the left ANG, right SMG, and left MOG were 0.99 ± 0.01 (*P* < 0.01), 0.70 ± 0.09 (*P* = 0.04), and 0.58 ± 0.10 (*P* = 0.40), respectively. Figure 6 illustrates that, for abnormal FC values in these multiple brain regions, the optimal cutoff value was determined to be 0.89 for the FC value of the left ANG (sensitivity, 95%; specificity, 94%), 0.50 for the FC value of the right SMG (sensitivity, 79%; specificity, 71%), and 0.30 for the FC value of the left MOG (sensitivity, 47%; specificity, 82%).

## Discussion

Utilizing ICA, we observed FC alterations in multiple regions across three RSNs in thirty-six neurologically asymptomatic ESRD patients undergoing HD, impacting various cognitive domains including visual-spatial processing, short-term memory, language, attention, and executive function. Significant correlations were identified between FC values of several brain regions (specifically, the right SMG, the left ANG, and the left MOG) and neurocognitive scale scores in HD patients. Moreover, FC values of the right MCC exhibited significant correlations with serum calcium levels. ROC analysis revealed that altered FC values in the left ANG and the right SMG effectively discriminate between ESHD patients with and without cognitive impairment.

We identified abnormal FC regions within the DMN in the ESHD group, particularly involving the bilateral PCUN and inferior parietal lobule (IPL). IPL includes the ANG and SMG. Dysfunction within the DMN can lead to impairments in learning/memory retrieval, self-reference processing, external orientation consciousness, and emotion regulation, potentially affecting the VN, AN, and SMN [16]. Furthermore, the precuneus (PCUN) plays a pivotal role in orchestrating integrated self-awareness tasks, encompassing self-processing operations, visual-spatial processing, and episodic memory retrieval [17]. The ANG integrates multisensory inputs and is pivotal in a range of cognitive functions, spanning visual-spatial abilities, language comprehension, and memory retrieval [18]. Similarly, the SMG is associated with episodic memory encoding, short-term auditory memory, and processing [19]. Our findings are consistent with several previous studies [10, 20–23]. For instance, Chen et al. reported abnormal spontaneous activity in the DMN among hemodialysis patients, localized to the PCUN and IPL/ANG, using amplitude of low-frequency fluctuations (ALFFs) [22] and regional homogeneity analysis [23], respectively. Chen et al. [24] utilized degree centrality (DC) and seed-based FC analyses to demonstrate increased DC values in the left SMG among patients with ESRD and cognitive impairment. Additionally, Zhang et al. [20] utilized diffusion tensor imaging technology to identify alterations in fiber bundles connecting the posterior cingulate cortex and the PCUN to the bilateral IPL in ESRD patients following renal transplantation. Furthermore, they reported positive correlations between these changes and scores on the NCT-A. In summary, both our study and previous research indicate that abnormal activity in PCUN and IPL, including the ANG and SMG, are characteristic of brain spontaneous activities in patients undergoing HD. Furthermore, our results demonstrate negative correlations between increased FC values of the right SMG and the NCT-A score, as well as decreased FC values of the left ANG and the NCT-B score. ROC analysis of the FC values of the left ANG and right SMG suggests that both can accurately identify patients with cognitive impairment during early hemodialysis. These findings provide further evidence supporting the notion that increased FC of the SMG may reflect a compensatory mechanism for maintaining cognitive function, while decreased FC of the ANG may signify the progression of cognitive decline in patients with ESHD.

We also observed abnormal FC regions in the right MFG. In previous research, Chen et al. [24] reported a positive correlation between the DC values of the right superior frontal gyrus and neurocognitive scores in patients with ESRD and cognitive impairment. Additionally, Chang et al. [21], using graph theory analysis, identified numerous abnormal FC regions in the frontal gyrus and cingulate gyrus among patients with ESRD receiving peritoneal dialysis but without cognitive decline. We observed abnormal FC values in the MFG among HD patients, which contrasts with findings regarding the superior frontal gyrus reported in the aforementioned studies. Based on previous research [25], we speculate that this difference may be attributed to variations in treatment modalities (e.g., conventional treatment, hemodialysis, and peritoneal dialysis) or differences in disease severity. Melissa et al. [26] reported that patients undergoing HD are more susceptible to impairments in visual-spatial processing, deductive reasoning, executive function, and verbal skills compared to short-term memory. These findings align with our speculation regarding the observed differences in FC values. However, validation of our findings will require a study with a larger sample size.

We also found decreased FC in the left MOG, a finding rarely reported in previous studies. Only one relevant study by Harrell et al. [27] investigated functional MRI during a visual-spatial working memory task in children with CKD, identifying activations in several brain regions including the frontal, occipital, temporal, and cingulate cortices, which are crucial for visual working memory and visual-spatial processing. We think that there may be several possible explanations for FC abnormalities in the MOG of HD patients. Firstly, prior research [28] has demonstrated that individuals with end-stage renal disease (ESRD) or CKD are more susceptible to visual impairment and eye diseases, suggesting a potential link between abnormal occipital lobe FC and visual impairment in HD patients. Secondly, dysfunction in the DMN connections could lead to abnormalities in visual, attentional, and sensorimotor networks, impacting feedback mechanisms within the frontoparietal and occipital cortices [29]. Thirdly, the MOG is involved in various functions including vision, language, executive function, and visual-spatial processing, all of which are areas vulnerable to cognitive impairment during HD [30]. Therefore, we speculate that intrinsic FC changes in the MOG may contribute to the cognitive decline observed in HD patients. Moreover, our speculation is supported by positive correlations between MoCA scores and decreased FC values in the left MOG, suggesting that FC changes in the MOG may serve as a signature of cognitive decline progression.

We also found a negative correlation between the increased FC value of the right MCC and serum calcium levels. Liu et al. [11] observed that changes in the white matter structure of the left anterior thalamic radiation were positively correlated with serum calcium levels. Given that hypocalcemia is common in patients with HD [31], it may induce neurotoxicity, neurotransmitter imbalance, and excessive parathyroid hormone stimulation, alongside 1,25(OH)2D3 deficiency [32]. This deficiency can lead to neurotoxicity affecting regions like the cingulate and prefrontal cortex. The MCC serves as a critical hub connecting the anterior and posterior cingulate cortices within the limbic system, playing pivotal roles in various cognitive functions [33, 34]. Although the MCC exhibits functional interactions with the DMN, its primary role is more closely associated with cognitive control and emotion regulation rather than being a core component of the DMN. Hence, we propose that hypocalcemia may directly or indirectly influence abnormal changes in MCC spontaneous activity in patients with ESHD, potentially impacting cognitive function through alterations in FC of the MCC.

Our research has several limitations. Firstly, being a cross-sectional study, we did not investigate whether the relationship between HD and cognitive impairment varies with dialysis duration; longitudinal research may offer insights in this regard. Secondly, the sample size of our study might limit the generalizability of our findings. Furthermore, our results do not account for the influence of dialysis parameters such as ultrafiltration rate, or the impact of primary diseases and associated complications in HD patients (e.g., hypertension, hypotension, transient ischemic attack, diabetes, parathyroid or thyroid hormone levels, depression, and anxiety). Lastly, in ESHD patients, the types and timing of neurocognitive scales used in our study may have influenced the sensitivity of different brain regions to cognitive function evaluation.

## Conclusion

In conclusion, this study highlights that early-stage patients with ESHD already exhibit multiple abnormal FC regions in three RSNs (the pDMN, AN, and EVN) using ICA technique, implicating visual-spatial processing, short-term memory, language, attention, and executive function in patients with HD within the first year of dialysis. These alterations may underlie cognitive dysfunction during the early HD process. Our results offer empirical evidence, suggesting that the observed clinical symptoms in the ESHD group are associated with FC changes. Importantly, our findings suggest that altered FC patterns hold promise for effectively identifying early-stage patients with ESHD with cognitive impairment. Additionally, the relationship between FC changes and serum calcium levels opens new avenues for targeted interventions, potentially mitigating cognitive decline in this vulnerable population.

## Data Availability

The datasets used during the current study are available from the corresponding author on reasonable request.

## Abbreviations

CKD: Chronic kidney disease
ESRD: End-stage renal disease
ESHD: Neurologically asymptomatic patients with ESRD undergoing maintenance hemodialysis
CI: Cognitive impairment
HC: Healthy control
HD: Hemodialysis
ICA: Independent component analysis
RNS: Abnormal resting-state networks
FC: Functional connectivity
NCT-A: Digital connections test types A
NCT-B: Digital connections test types B
LTT: Line-tracing test
SDT: Serial dot test
DST: Digit symbol test
MoCA: The Montreal cognitive assessment
DMN: Default mode network
VN: Visual network
LVN: Lateral visual network
EVN: External visual network
SMN: Sensorimotor network
AN: Attention network
SMG: Supramarginal gyrus
MCC: Median cingulate gyrus
ANG: Angular gyrus
PCUN: Precuneus gyrus
MFG: Middle frontal gyrus
MOG: Middle occipital gyrus
